# High-Grade Glioma Mimicking Eclampsia in Pregnancy: A Case Report

**DOI:** 10.7759/cureus.117087

**Published:** 2026-09-28

**Authors:** Sutharsika Karthic

**Affiliations:** 1 Obstetrics and Gynaecology, University Hospitals of Leicester NHS Trust, Leicester, GBR; 2 Obstetrics and Gynaecology, Prashanth Super Speciality Hospital, Chennai, IND

**Keywords:** eclampsia mimic, eclampsia with pres, haemorrhagic brain lesion, high-grade glioma in pregnancy, hypertensive encephalopathy, intracranial tumour, neuroimaging in pregnancy, posterior reversible encephalopathy syndrome (pres), preeclampsia-eclampsia, seizures during pregnancy

## Abstract

Eclampsia is the most common cause of new-onset seizures after 20 weeks of gestation and requires immediate treatment. However, not all seizures in pregnancy are caused by hypertensive disorders, and structural intracranial lesions may present with similar clinical features.

A 39-year-old primigravida at 25+6 weeks' gestation presented to Prashanth Super Speciality Hospital with severe hypertension (200/100 mmHg), proteinuria, altered sensorium, and recurrent generalized tonic-clonic seizures. She was initially managed as eclampsia with suspected posterior reversible encephalopathy syndrome (PRES). Owing to maternal instability, an emergency lower-segment caesarean section was performed. Persistent neurological impairment after delivery prompted neuroimaging. Computed tomography demonstrated a left frontal intracerebral haemorrhage, while magnetic resonance imaging revealed a haemorrhagic left frontal mass with surrounding oedema, midline shift, and PRES-like imaging features. Following urgent neurosurgical intervention, the patient underwent left frontal craniotomy and complete tumour excision. Histopathological examination confirmed a high-grade glioma. The patient recovered well postoperatively and was referred to the neuro-oncology team and subsequently underwent adjuvant radiotherapy and chemotherapy.

The neonate was delivered at 25+6 weeks' gestation and died on day 2 of life despite neonatal intensive care.

This case demonstrates that structural brain tumours may closely mimic eclampsia, even in the presence of severe hypertension and proteinuria. Persistent neurological abnormalities following standard treatment for eclampsia should prompt urgent neuroimaging to exclude underlying intracranial pathology. Early multidisciplinary collaboration between obstetricians, critical care specialists, radiologists, neurologists, neurosurgeons, neonatologists, and oncologists is essential to optimise maternal outcomes.

## Introduction

Seizures during pregnancy represent a potentially life-threatening obstetric emergency requiring prompt assessment and management. Eclampsia remains one of the most important causes of new-onset seizures after 20 weeks of gestation and is classically associated with hypertension and proteinuria. Early recognition and treatment with magnesium sulphate, antihypertensive therapy, and timely delivery when indicated are essential to reduce maternal and fetal morbidity and mortality [1].

However, seizures in pregnancy are not exclusively caused by hypertensive disorders, and clinicians must maintain a broad differential diagnosis, particularly when neurological recovery is delayed or atypical. Neurological conditions that may present with seizures during pregnancy and the puerperium include posterior reversible encephalopathy syndrome (PRES), cerebral venous sinus thrombosis, intracerebral haemorrhage, epilepsy, central nervous system infections, metabolic disturbances, and intracranial space-occupying lesions [2,3]. The clinical overlap between eclampsia, PRES, and intracranial pathology can make early diagnosis challenging, particularly when severe hypertension and seizures occur together. Delayed recognition of an underlying neurological disorder may result in progressive neurological deterioration and adverse maternal and fetal outcomes.

PRES is a well-recognised neurological complication associated with severe preeclampsia and eclampsia. It occurs due to endothelial dysfunction and failure of cerebral autoregulation, resulting in vasogenic cerebral oedema, or reversible brain swelling caused by disruption of normal cerebral blood-flow regulation. MRI typically demonstrates bilateral posterior predominant vasogenic oedema; however, atypical imaging findings may occur, including haemorrhage, extensive oedema, and focal abnormalities [3-6]. Therefore, radiological findings suggestive of PRES should be interpreted in the context of the overall clinical picture, particularly when neurological symptoms persist despite appropriate treatment. Other neurological causes of seizures in pregnancy, including cerebral venous sinus thrombosis, should also be considered, particularly when symptoms persist or do not follow the expected course [7].

Primary brain tumours diagnosed during pregnancy are rare but can present with symptoms that overlap with common pregnancy-related conditions, including headache, vomiting, seizures, and altered mental status. Although high-grade gliomas are uncommon during pregnancy, delayed diagnosis may result in progressive neurological deterioration and can complicate decisions regarding maternal treatment and fetal wellbeing. These similarities can contribute to diagnostic delay, especially when symptoms occur alongside hypertensive disorders of pregnancy [8,9]. High-grade gliomas are particularly challenging because they require balancing maternal neurological needs, fetal considerations, timing of delivery, and the need for neurosurgical intervention [9,10].

The physiological changes associated with pregnancy, including hormonal, vascular, and immunological alterations, may influence tumour growth and clinical presentation, although the exact mechanisms remain uncertain [9]. Management of high-grade gliomas during pregnancy is complex and requires multidisciplinary collaboration between obstetricians, neurosurgeons, oncologists, radiologists, anaesthetists, and neonatal teams. Current evidence is limited, with most available data derived from case reports and small case series [10].

Neuroimaging is essential when neurological symptoms persist or do not follow the expected course after treatment for presumed eclampsia. MRI provides excellent soft tissue characterisation and is considered safe when clinically indicated during pregnancy [11]. Early identification of intracranial pathology allows appropriate intervention and may improve maternal outcomes.

We present an uncommon case of a high-grade glioma manifesting with features of eclampsia in a 39-year-old primigravida at 25+6 weeks’ gestation. The patient presented with severe hypertension, proteinuria, altered consciousness, and recurrent seizures, initially managed as eclampsia with suspected PRES. Persistent neurological impairment following delivery led to further investigation, revealing a haemorrhagic frontal lobe tumour with PRES-like imaging features. This case highlights the importance of maintaining diagnostic vigilance and considering alternative neurological diagnoses when the clinical course is atypical.

## Case presentation

A 39-year-old primigravida with a spontaneous conception presented at 25+6 weeks’ gestation to Prashanth Super Speciality Hospital with severe hypertension, altered sensorium, and recurrent seizures. She had received antenatal care at another hospital, where persistent hypertension and proteinuria had previously been documented. Despite medical advice, she had declined antihypertensive treatment. Her past medical history was significant only for hypothyroidism. There was no history of epilepsy, previous neurological disease, significant surgical history, or family history of neurological disorders.

She was brought to the emergency department with a blood pressure of 200/100 mmHg, reduced consciousness, and two episodes of generalised tonic-clonic seizures, one occurring at home and another shortly after arrival. Tongue biting and oral bleeding supported the diagnosis of true seizure activity. No focal neurological deficits were identified on initial examination. Initial laboratory investigations, including serum electrolytes and blood glucose, were unremarkable. In view of the presence of severe hypertension, proteinuria, and seizures occurring after 20 weeks of gestation, a provisional diagnosis of eclampsia with PRES was made.

Initial management included seizure control with intravenous magnesium sulphate, antihypertensive therapy, and supportive care. Obstetric assessment confirmed a uterus corresponding to approximately 26 weeks’ gestation, with fetal heart activity present initially. Due to maternal instability and the need for urgent delivery, an emergency lower-segment caesarean section was performed on 4 February 2023.

A live female infant weighing 435 g was delivered in poor condition. Meconium-stained liquor was noted, and the neonate required immediate resuscitation before admission to the neonatal intensive care unit. Despite neonatal intensive care, the infant died on day 2 of life.

During surgery, moderate ascites was observed. A 2 × 2 cm subserosal fibroid and a 5 × 6 cm intramural fibroid were identified. Both ovaries and fallopian tubes appeared normal. An intraperitoneal drain was inserted, and estimated blood loss was within normal limits.

Following delivery, the patient was transferred intubated to the high-dependency unit for ongoing management. She received intravenous antihypertensive therapy, magnesium sulphate infusion for seizure prophylaxis, ventilatory support, and broad-spectrum antibiotics. Despite appropriate management of presumed eclampsia, her neurological status failed to improve as expected following delivery. Persistent reduced consciousness raised concern for an alternative or coexisting neurological pathology.

A non-contrast computed tomography (CT) scan of the brain demonstrated a left frontal intracerebral haemorrhage, prompting consideration of cerebral venous sinus thrombosis or a haemorrhagic space-occupying lesion. Contrast-enhanced MRI subsequently revealed a left frontal haemorrhagic mass with surrounding oedema, significant midline shift, and imaging features suggestive of PRES (Figure 1). The coexistence of a haemorrhagic tumour and PRES-like changes complicated the diagnostic assessment and necessitated urgent neurosurgical review.

**Figure 1:**
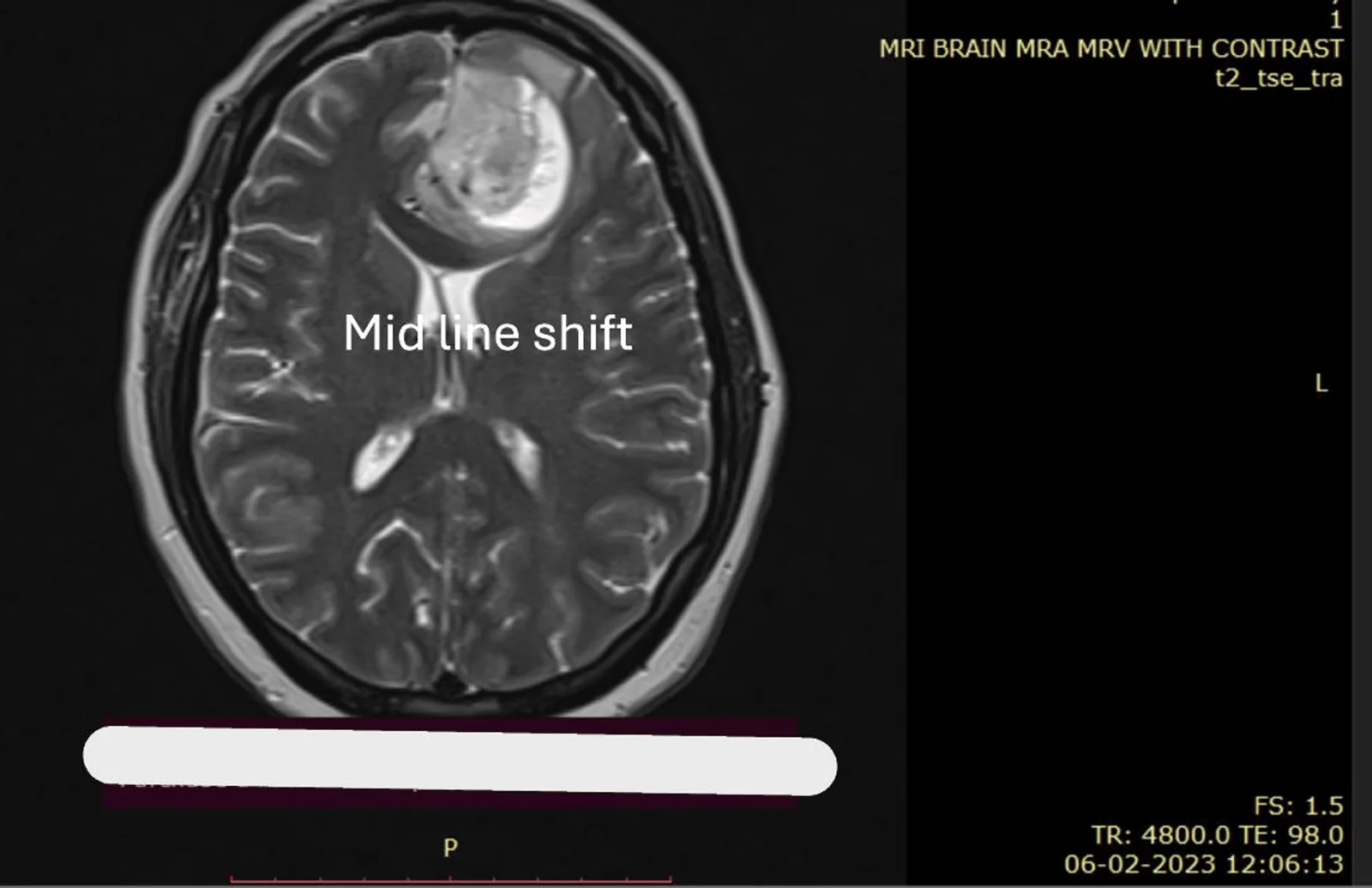
Contrast-enhanced MRI of the brain demonstrating a haemorrhagic left frontal lobe mass with surrounding vasogenic oedema and significant midline shift, consistent with a space-occupying lesion presenting with PRES-like imaging features. PRES: Posterior reversible encephalopathy syndrome

Electroencephalography and transthoracic echocardiography were unremarkable. A multidisciplinary team discussion involving obstetrics, neurology, neurosurgery, radiology, anaesthesia, and critical care teams was undertaken. Given the significant mass effect and neurological deterioration, the patient underwent an urgent left frontal craniotomy with complete tumour excision on 6 February 2023.

Histopathological examination confirmed a diagnosis of high-grade glioma. Following surgery, the patient demonstrated progressive neurological improvement and was successfully extubated on 7 February 2023. Follow-up CT imaging demonstrated satisfactory postoperative appearance without evidence of a residual tumour (Figure 2). She was subsequently referred to the neuro-oncology team for further management and underwent adjuvant radiotherapy and chemotherapy.

**Figure 2:**
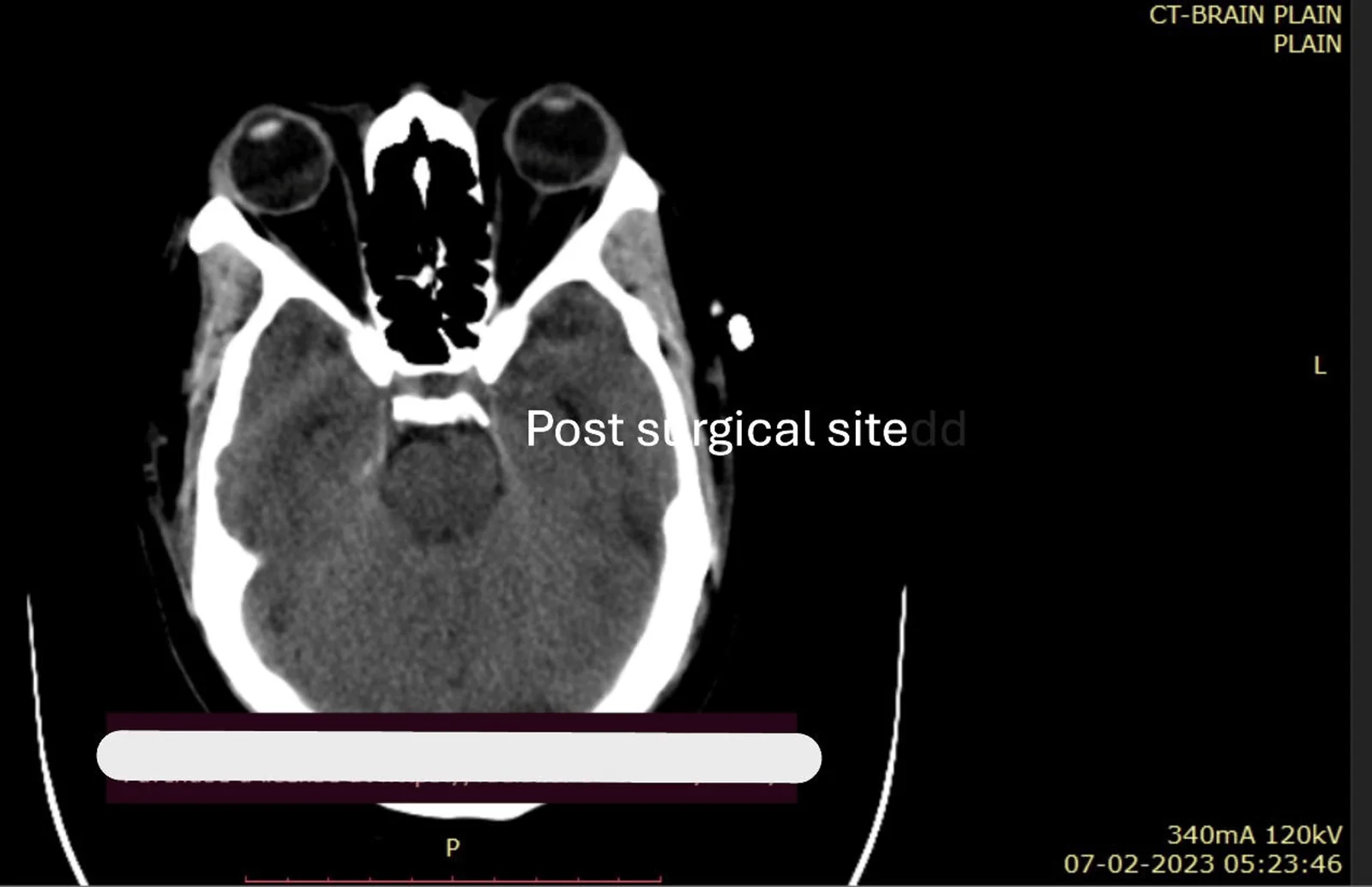
Postoperative non-contrast CT brain demonstrating satisfactory postoperative changes following left frontal craniotomy and tumour excision, with no evidence of a residual mass lesion.

This case demonstrates the diagnostic complexity of seizures in pregnancy, particularly when clinical features overlap with eclampsia and PRES. Although the initial management for eclampsia was appropriate, persistent neurological dysfunction after delivery prompted further investigation, leading to the diagnosis of an underlying intracranial tumour.

## Discussion

This case highlights the diagnostic challenge of differentiating eclampsia from underlying intracranial pathology during pregnancy. The patient presented with severe hypertension, proteinuria, altered consciousness, and recurrent generalised seizures after 20 weeks of gestation, making eclampsia the most appropriate initial diagnosis. Prompt management with magnesium sulphate, blood pressure control, and expedited delivery was clinically justified. However, the persistence of neurological impairment following delivery suggested that an alternative or additional neurological diagnosis needed to be considered.

Although eclampsia remains one of the most important causes of seizures in pregnancy, clinicians should maintain a broad differential diagnosis, particularly when the clinical course is atypical or neurological recovery is delayed [1-3]. Neurological conditions including epilepsy, central nervous system infections, metabolic disorders, and intracranial tumours may present with similar symptoms and can coexist with hypertensive disorders of pregnancy [2,3]. Cerebral venous sinus thrombosis is an important neurological differential diagnosis during pregnancy and the puerperium because it may present with seizures, altered consciousness, and other neurological symptoms [7]. Failure to recognise alternative diagnoses may result in delayed treatment and increased maternal morbidity.

PRES was an important consideration in this patient due to the presence of severe hypertension, seizures, altered consciousness, and MRI findings suggestive of vasogenic cerebral oedema. PRES is a recognised complication of severe preeclampsia and eclampsia, resulting from endothelial dysfunction and impaired cerebral autoregulation [3,4]. Typical clinical features include seizures, headache, visual disturbances, and altered mental status [4]. MRI usually demonstrates bilateral posterior-predominant oedema [5,6]. However, PRES may demonstrate atypical imaging findings, including haemorrhage, extensive oedema, and focal abnormalities. In this case, although PRES-like changes were present, the identification of a haemorrhagic frontal mass with significant midline shift indicated an underlying structural lesion rather than isolated PRES.

The coexistence of tumour-related changes and PRES-like imaging findings created an additional diagnostic challenge. This emphasises the importance of interpreting radiological findings within the clinical context and avoiding premature diagnostic closure. Persistent neurological impairment despite appropriate treatment for eclampsia should prompt urgent neuroimaging to exclude intracranial pathology.

High-grade gliomas diagnosed during pregnancy are rare, and diagnosis can be delayed because presenting symptoms often overlap with common pregnancy-related complaints. Headache, nausea, vomiting, seizures, and altered mental status may initially be attributed to pregnancy, preeclampsia, or other obstetric conditions [8-10]. The physiological changes of pregnancy, including hormonal, vascular, and immune modulation, may influence tumour behaviour, although the exact mechanisms remain unclear [9].

Management of high-grade gliomas during pregnancy requires careful consideration of multiple factors, including maternal neurological status, gestational age, fetal viability, tumour characteristics, and timing of intervention [9,10]. Available evidence is limited, and treatment decisions are often individualised through multidisciplinary discussion. Advances in molecular classification have improved understanding of glioma biology and have influenced modern treatment strategies, including personalised approaches to surgery, radiotherapy, and systemic therapies [12,13]. Following complete surgical excision, the patient was subsequently referred to the neuro-oncology team and underwent adjuvant radiotherapy and chemotherapy.

Neuroimaging played a pivotal role in establishing the diagnosis in this case. Computed tomography provided rapid identification of intracranial haemorrhage, while MRI allowed better characterisation of the underlying lesion and demonstrated the extent of oedema and mass effect. MRI is considered an appropriate investigation when clinically indicated during pregnancy, with current evidence supporting its safety [11]. A low threshold for neuroimaging should therefore be maintained in pregnant patients with seizures or altered consciousness that do not resolve as expected after standard eclampsia management.

The management of this patient required close collaboration between obstetrics, critical care, radiology, neurology, neurosurgery, neonatology, and oncology teams. Multidisciplinary decision-making is particularly important in rare clinical scenarios where maternal and fetal considerations must be balanced alongside urgent neurological treatment. Current management principles for severe preeclampsia and eclampsia emphasise early recognition, seizure prevention with magnesium sulphate, blood pressure control, and timely delivery when clinically indicated [14].

The main learning point from this case is that eclampsia should be treated promptly when suspected; however, clinicians must remain vigilant for alternative diagnoses when the clinical course is atypical. Persistent altered consciousness, recurrent seizures, unexpected neurological deterioration, or abnormal imaging findings should prompt evaluation for underlying intracranial pathology. Early diagnosis and timely intervention may significantly improve maternal outcomes.

## Conclusions

High-grade gliomas can rarely present during pregnancy with clinical features that closely mimic eclampsia, including hypertension, proteinuria, and seizures. Although eclampsia remains the most likely diagnosis in such presentations, persistent neurological abnormalities after appropriate treatment should prompt consideration of alternative intracranial causes.

This case emphasises the importance of maintaining a broad differential diagnosis, early neuroimaging when recovery is not as expected, and effective multidisciplinary collaboration. Recognising atypical presentations may facilitate timely diagnosis, appropriate neurosurgical management, and improved maternal outcomes.
